# Measuring Gender Role Conflict, Internalized Stigma, and Racial and Sexual Identity in Behaviorally Bisexual Black Men

**DOI:** 10.1007/s10508-021-01925-w

**Published:** 2021-06-16

**Authors:** Homero E. del Pino, W. Neil Steers, Martin Lee, Jason McCuller, Ron D. Hays, Nina T. Harawa

**Affiliations:** 1 Department of Psychiatry and Human Behavior, Charles R. Drew University of Medicine and Science, Los Angeles, CA USA; 2 Department of Medicine, David Geffen School of Medicine at UCLA, Suite 850, 1100 Glendon Ave., Los Angeles, CA 90024 USA; 3 Department of Biostatistics, UCLA Fielding School of Public Health, Los Angeles, CA USA; 4 College of Medicine, Charles R. Drew University of Medicine and Science, Los Angeles, CA USA

**Keywords:** Black MSMW, Race, Gender role conflict, Intersectional identity, Internalized stigma, Sexual orientation

## Abstract

Black men who have sex with men and women (BMSMW) experience pressure to fill hypermasculine ideals and may not identify with “gay” cultural norms. Existing measures of gender role expectations and internalized homophobia are not culturally appropriate for BMSMW. Researchers generally measure categorical identification with race, gender, and sexual orientation groups separately, whereas BMSMW may identify with multiple categories. We modified the Gender Role Conflict Scale to create the M-GRCS and the Internalized Homophobia Scale to include biphobia (Internalized Bi/Homophobia Scale, IBHS). To examine identification at the intersection of race, gender, and sexual orientation, we created 11 Integrated Race and Sexuality Scale (IRSS) items. With data from 429 BMSMW, we conducted exploratory factor analysis of the 59 items using categorical principal axis factoring with unweighted least squares extraction and Promax factor rotation. We created simple-summated multi-item scales and evaluated their construct validity. The rotated solution yielded four factors with 47 items and a simple factor structure: M-GRCS defined two factors (*α* = .93 for restricted emotionality/affection; .87 for success/power/competition); the IBHS (*α* = .89) and IRSS (*α* = .74) each defined a single factor. The IRSS factor was positively correlated with the Lukwago Racial Pride Scale, *r*(417) = .40. The IBHS factor was negatively correlated with the IRSS factor, *r*(414) = − .22. The two M-GRCS factors suggest that the construct of hypermasculinity impacts BMSMW. The high IBHS reliability indicates that homophobia and biphobia were positively correlated in this sample. These three scales have potential for future studies with BMSMW.

## Introduction

Gender role conflict, hypermasculinity, and internalized homophobia have negative effects on the health-related risk behaviors and healthcare engagement of Black men in the U.S. who have sex with both men and women (BMSMW). Traditional masculine roles prescribe behaviors such as “Be powerful” and “Do not show fear, be feminine or display affection toward other men” (Hall & Applewhite, 2013; Ward, 2005). Hypermasculinity, an exaggeration of traditional masculine traits, stresses physical strength, aggression, dominance, and sexual prowess, and a man lacking these characteristics is considered weak and feminine (LaPollo, Bond, & Lauby, 2014; Ward, 2005). In a study of men who have sex with men and women (MSMW) (*n* = 281, Black, 64%, and white, 36%), LaPollo et al. (2014) found hypermasculine ideals to be associated with income, ever being in jail, feeling that it is “very important” to keep MSM behavior secret, and greater internalized homophobia. Some have suggested that “Black masculinities” evolved in response to the emasculation of Black men during slavery and ongoing racism that includes violence, educational and economic deprivation, and sexualization, resulting in hypermasculine prescriptions for behavior (LaPollo et al., 2014; Ward, 2005). Similarly, some have argued that hypermasculinity has been embraced among gay men in response to subordination by a dominant heterosexual male culture (Fields et al., 2015). Both types of masculinity, Black masculinity and hypermasculinity, have also been referred to as “compensatory masculinity” because they are formed “in reaction to blocked access to power and authority” (Fields et al., 2015).

Others point out that researchers have ignored aspects of Black men’s masculinity, such as self-determinism, accountability, family, community, pride, and spirituality, that have helped to maintain Black families and communities over time (Hammond & Mattis, 2005; Hunter & Davis, 1992, 1994; Wade & Rochlen, 2013). Arguably, these positive aspects of Black men’s masculinity have been even more overlooked in reference to Black MSMW. The focus instead has been on how Black men’s socioeconomic status, gender role expectations, and racial stratification may increase motivations for sex with women among men who are attracted to both men and women or only men (Bowleg et al., 2011; Carey, Senn, Seward, & Vanable, 2010; Nunn et al., 2011). This focus on negative aspects may be because of how traditional masculine gender role expectations are associated with health-comprising behaviors and beliefs, such as having multiple sex partners, resistance to using condoms, declining services such as human immunodeficiency virus (HIV) testing, as well as holding negative attitudes toward both women and men who have sex with men (MSM) (Hall & Applewhite, 2013; LaPollo et al., 2014; Mankowski & Maton, 2010; Rhodes et al., 2011). Feeling pressured to live up to masculine or hypermasculine ideals has also been linked with internalized homonegativity (Estrada, Rigali-Oiler, Arciniega, & Tracey, 2011; Miller, 2015). Although hypermasculinity is associated with a higher number of both male and female partners among BMSMW, it appears to have a stronger influence on the number of female partners than male partners (LaPollo et al., 2014).

The role of socialization in masculine gender role expectations has also been overlooked. Black men are subject to different socialization processes than White men (e.g., racial discrimination, economic exclusion); these different social realities lead to different constructions of masculinity (Wade & Rochlen, 2013). Racial differences in the construction and expectations of masculine gender roles might explain differences in behavior between Black and White MSMW. For Black MSMW, hypermasculinity and internalized homophobia are significantly associated with a greater number of recent male and female sex partners, whereas for White MSMW they are associated only with a higher number of female sex partners (LaPollo et al., 2014). Similarly, differences in how masculine gender roles impact those whose behaviors and/or identities reflect attraction to both men and women versus only men might also explain differences in behavior between Black MSMW and Black MSM only. For example, Black MSMW may experience more pressure than do Black MSM to uphold traditional Black family values and fill the role of partners to Black women. Compared with Black MSM, Black MSMW engage in more transactional sex, insertive anal intercourse, substance use, and have more criminal justice involvement; they also report experiencing more physical assaults, intimate partner violence, and depressive symptoms (Dyer et al., 2013; Friedman et al., 2019a; LaPollo et al., 2014). Among gay- and bisexually identified men (of which almost 30% were Black), Feinstein, Moran, Newcomb, and Mustanski (2019) found that bisexually identified men reported more condomless insertive anal sex with casual partners than did gay-identified men. They also reported more frequent marijuana use and lower levels of lifetime HIV testing and pre-exposure prophylaxis (PrEP) use. Another study found that Black MSMW were less likely to report PrEP awareness than were Black MSM; however, among those who were aware of PrEP, Black MSMW were more likely to actually use it than were Black MSM (Friedman et al., 2019b). Finally, MSMW of all race/ethnicities tend to have lower socioeconomic status than do MSM (Shadaker, Magee, Paz-Bailey, Hoots, & NHBS Study Group, 2017), which may foster compensatory masculinity.

Researchers have often grouped Black MSMW with Black MSM or with White MSMW, even though their sexual behaviors, HIV-related risks, racial and sexual identities, and self-perceptions differ. Researchers use measures, outreach strategies, and interventions that have been developed specifically for MSM and/or developed primarily with White MSMW, all of which often explicitly or implicitly assume monosexual behaviors and gay or bisexual identities. For example, existing measures of internalized homophobia for MSM tend to use terms like “gay” and assume that respondents are primarily attracted to men (Mayfield, 2001). This may affect the responses of MSMW for reasons unrelated to their attitudes regarding sex between men. In addition, some Black men have criticized and rejected the term “gay” for cultural and historical reasons, arguing that it has homophobic and Eurocentric origins (Parks, 2001).

New measures, focused on biphobia, have been created since we conducted our research. One new measure of anti-bisexual discrimination was developed with only 15 (4%) of the 422 participants identifying as Black (compared with 345 [82%] who identified as White) (Dyar, Feinstein, & Davila, 2019), and a brief version of the Anti-Bisexual Experiences Scale was administered to 390 participants, of whom only 9 (2.3%) identified as “African American/Caribbean American/Black” (Paul, Smith, Mohr, & Ross, 2014). Blacks comprised 33% of the sample with which the Negative Attitudes Toward Same-Sex Behavior Inventory was developed and validated, yet it did not include race as a domain (Antebi-Gruszka & Schrimshaw, 2018). The small number of Black MSMW included in the development and evaluation of these measures, as well as not including race as a domain, calls into question these instruments’ cultural relevance and suitability for Black MSMW. Research to date hints at the limitations of using the same instrument—either with both MSMW and MSM (regardless of race) or with racially diverse participants (regardless of sexual identity)—to measure a construct that is shaped by each group’s race, culture, and sexual identity (Miller, 2015).

Too often research approaches and survey measures overlook Black men’s multiple and intersecting identities (e.g., race, class, sexual identity) and how these identities reflect inequalities at the sociostructural level, that is, those produced by racism and heterosexism (Bowleg et al., 2017). This is due in part to the fact that a majority of studies on Black men’s sexuality have been designed, conducted, analyzed, and interpreted by researchers who were not Black, which often led to the construction of Black sexuality as hypermasculine, hyperheterosexual, aggressive, and influenced by epistemologies of ignorance that are rooted in racism (Bowleg et al., 2017; Ford, Whetten, Hall, Kaufman, & Thrasher, 2007). An intersectional framework emphasizes that race, gender, class, and sexual identity cannot be extricated from each other, that these identities reinforce each other, and that a person’s social location, such as that determined by racialized processes, cannot be properly understood without accounting for how it intersects with other key locations, such as sexual identity (Bowleg et al., 2017). In the case of Black MSMW, it is important to recognize the complexity of the heteronormative environments within which they live. Their stigmatized sexual statuses, their sometimes-threatened and questioned masculinity, their racial designations, and the high levels of poverty they face cannot be reduced to discrete experiences or identities or analyzed along a single axis (e.g., sexuality). Ignoring key aspects of Black MSMW’s experiences constrains our capacity to understand how they self-identify and how they respond to the expectations of traditional masculinity and heteronormativity.

While developing and testing a holistic HIV risk-reduction intervention for Black MSMW (Harawa et al., 2013), we sought to reduce the negative impact of gender roles on HIV risk and preventive behaviors and healthcare seeking. Prior efforts to address these behaviors among Black men and Black MSMW had considered neither the interrelated experiences of race, class, and “compensatory masculinity,” nor the nontraditional aspects of masculinity valued by Black men. Furthermore, sound measures of biphobia were not available at the time of our research nor were measures that assessed Black MSMW’s overlapping experiences and identities related to race, gender, and sexual orientation. In order to address these gaps, we used an iterative, community-informed process: (1) to modify existing measures of internalized homophobia and gender role conflict for Black MSMW and (2) to develop a measure of integrated racial and sexual identities. Below, we describe each of these measures, the rationale for specific adaptations, the psychometric properties of these scales, and their construct validity.

## Method

### Development of Measures

Our primary objective was to assess mediators of sexual risk behaviors for an HIV prevention intervention study of low-income Black MSMW (Williams, Ramamurthi, Manago, & Harawa, 2009). We were also interested in exploring associations that had not been adequately studied in this subgroup. The intervention was designed to reduce the frequency of condomless sex and the number of sex partners and to increase HIV testing among Black MSMW (Harawa et al., 2013).

To develop the study’s survey instrument, the investigative team first identified measures of interest for our selected domains. These domains were informed by the behavioral models on which our intervention was based and our formative research with the study population, which included focus groups and one-on-one interviews (Harawa, Williams, Ramamurthi, & Bingham, 2006; Harawa et al., 2008). We then selected from the literature those measures that most closely captured these latent constructs and that had been evaluated with and shown validity in samples of Black adults in the U.S. Next, we reviewed the measures with a community advisory board comprised of Black MSM and MSMW and with community partners who had extensive experience serving these two subgroups. Participants suggested wording modifications and, in some cases, identified items that they perceived to be irrelevant or suggested new items. As a result of this process, we modified two existing scales, the GRCS and the Internalize Homophobia Scale, and wrote new items for a third scale, which we named the Integrated Race and Sexuality Scale. Next, we conducted cognitive interviews with 20 Black MSMW to ensure the modifications and new scale items were clear, easy to understand, and related to the concepts of interest. Minor additional modifications were made based on feedback from this process. These three scales are described below.

### Modified Gender Role Conflict Scale (M-GRCS)

The original GRCS was administered using a 1 (strongly disagree) to 6 (strongly agree) response scale (O’Neil, Helms, Gable, David, & Wrightsman, 1986). We deleted seven of the original 37 items and added five new items, resulting in 35 items. The added items were designed to measure the importance of masculine presentation (described below) to participants. Six of the deleted items were from the conflict between work and family relations subscale (e.g., “My work or school often disrupts other parts of my life [home, health, leisure]”), which was deemed to lack relevance for our group of largely unemployed/underemployed men. Szymanski and Carr (2008) also noted concerns about the validity of these items. The seventh item, “Telling my partner my feelings about him or her during sex is difficult for me,” was deleted because our community advisory board and community partners indicated that it seemed repetitive and might lack relevance if individuals were engaging in sex for reasons unrelated to any kind of emotional attachment, such as exchange sex or anonymous sex. Additionally, we made minor wording changes to eight items to improve their clarity and relevance to the target population. This modified version of the GRCS, the M-GRCS, measures participants’ view of gender role stereotypes in four main areas (Bingham, Harawa, & Williams, 2013): (1) success, power, and competition; (2) difficulty expressing emotions or having others express them; (3) difficulty showing or observing affection between men; and (4) masculine presentation (e.g., “Men should never show their feminine side” and “I never want to look or seem weak”).

### Internalized Bi/Homophobia Scale (IBHS)

The Internalized Homophobia Scale (IHS) has nine items (Martin & Dean, 1987). The IHS was administered using five response options, from 1 (strongly disagree) to 5 (strongly agree). While the scale has been shown to have acceptable reliability (*α* > 0.70), there is limited information about its factor structure. An unpublished analysis suggested a single factor for the IHS items (Herek, Gillis, Cogan, & Glunt, 1997). Although the IHS has been administered in studies with gay-, lesbian-, and bisexual-identified populations, BMSMW comprised a small portion of the study samples (Martin & Dean, 1987). The IHS was administered to a sample of 741 gay-identified men in New York City, but only 11% were men of color (Meyer, 1995). Herek et al. (1997) evaluated a gender-specific version of the scale in a sample of 73 men and 74 women recruited from a lesbian, gay, and bisexual street fair in Sacramento of which 18% were people of color, 14% self-identified as bisexual, and the rest identified as lesbian or gay.

We modified the wording of the nine original IHS items because our study participants were men who reported sex with both men and women, generally were attracted to both men and women, did not necessarily identify as gay or see themselves as part of the gay community, and identified with a range of labels for their sexuality. We also added four items suggested by our community experts either to clarify responses to original questions or to ask specifically about bisexual identification and behavior. We called the new 13-item scale the Internalized Bi/Homophobia Scale (IBHS).

### Integrated Race and Sex Scale (IRSS)

This new scale has 11 items assessing the intersecting levels of internalized pride and conflict regarding participants’ status as male members of minority groups defined by sexual behavior and race. IRSS uses a 1 (strongly disagree) to 5 (strongly agree) response scale, selected to allow for a neutral option. Four items examined the perceived role of Black homosexual and bisexual men in communities and families (e.g., “Black homosexual and bisexual men contribute to Black communities”). Two items examined perceptions of Black male relationships (e.g., “I cannot imagine a loving sexual relationship between two Black men”), and one item explored whether a Black MSM can fulfill other masculine expectations: “A Black man who has sex with men can still be a strong man.” Three items examined how participants wish to be seen in relation to their gender, race, and sexual orientation (e.g., “It is more important that people see me as a man than as a heterosexual man”), and one item explored the importance of race and sexuality in relation to participants’ gender identity: “Both my race and my sexuality are important to who I am as a man.”

We developed this scale to move beyond items used in prior research that assessed the relative value participants placed on their racial and sexual identities, including those measures that asked participants to rank the relative importance of these identities and affiliations. Specific items further assessed our premise about the centrality of masculine gender roles to participants’ self-perceptions and interactions with others. In addition, the scale was developed to assess whether the intervention led to a stronger and more integrated sense of oneself in relation to these categories.

### Analysis

We conducted an exploratory factor analysis of the M-GRCS, IBHS, and IRSS items using categorical principal axis factoring with unweighted least squares (ULS) extraction and Promax factor rotation (Hendrickson & White, 1964). Three different factor criteria were examined: the scree plot, Guttman's weakest lower bound, and Guttman's third bound (Cattell, 1966; Guttman, 1940). The results of the factor analyses were used to create simple-summated multi-item scales. We estimated internal consistency reliability for the scales (coefficient alpha): ≥ 0.90 = excellent; 0.80-0.89 = good; and 0.70-0.79 = acceptable (Cronbach, 1951).

We evaluated the construct validity of the modified scales by estimating their correlations with “criterion” measures. We chose the 7-item Lukwago scale of racial pride (*α* = 0.84) as the criterion for the IRSS (Lukwago, Kreuter, Bucholtz, Holt, & Clark, 2001) and expected a positive association. We also hypothesized a negative association of the IRSS with the Restricted Emotionality and Discomfort with Affection between Men subscales from the M-GRCS. For the IBHS, we chose several items indicating whether or not a participant had disclosed his sexual orientation to each of several types of individuals, including family members, friends, and female sex partners. We also examined associations of the IBHS with the following: reports of numbers of one’s friends knowing one’s same-sex sexual orientation, the perceived importance of one’s friends knowing this, perceived importance of keeping one’s sexual relationships with men secret, and how often one had sex with a woman who did not know one’s sexual orientation, as additional criteria variables for the IBHS. We chose the IBHS as a criterion for the Restricted Emotionality, Discomfort with Affection between Men*,* and Success/Power/Competition subscales of the M-GRCS, hypothesizing that those with higher levels of bi/homophobia would report increased higher levels of these M-GRCS constructs.

We examined correlations of the scales with psychological distress (Brief Symptom Inventory-53) (Derogatis & Melisaratos, 1983) and self-esteem (10-item Rosenberg Self-Esteem Scale) (Rosenberg, 1979). We hypothesized that the IBHS and the M-GRCS would be associated with higher levels of distress and lower levels of self-esteem and that the IRSS would be associated with lower levels of distress and higher levels of self-esteem. Finally, because both gender role conflict and internalized bi/homophobia may reflect an internalization of perceived norms from one’s social networks, we examined associations with the friend and family subscales of the Multidimensional Scale of Perceived Social Support (Zimet, Dahlem, Zimet, & Farley, 1988).

## Results

A total of 429 respondents completed the items as part of their baseline (pre-intervention) self-administered (via computer) survey (see Table 1). Men aged 40–49 years were the largest age group (43%), followed by those over 50 years old (24%); those under 18 years of age comprised the smallest group (14%). The majority of the men self-identified as bisexual (61%), followed by heterosexual/straight/down low or DL (21%) and gay or homosexual (13%). Most of the men had a history of incarceration (*n* = 328, 77%). Although most participants (58%) had completed high school, a general equivalency diploma (GED), or higher educational level (26%), more than half (56%) reported earning less than $1,000 per month and almost half were unemployed (46%). Only 15% of the men reported currently living with a spouse or partner. Forty percent had experienced housing instability in the prior12 months. Nearly half of the participants had previously tested HIV-positive, 43% had last tested HIV-negative, and 8% had never tested.

**Table 1:** Participant characteristics (n = 429)

Variable	Frequency	Percent
Age		
18–29	62	14
30–39	78	18
40–49	186	43
50 +	103	24
Sexual orientation		
Heterosexual (straight), down low, or DL	92	21
Bisexual	263	61
Gay, homosexual or same gender loving or SGL	54	13
Other/none of the above	20	5
Which sex more frequently attracted to		
More often women than men	132	31
About the same	150	35
More often men than women	145	34
Highest level of education completed		
Less than high school	71	17
High school diploma or GED	247	58
Two-year associate degree or certificate	81	19
College or professional degree	30	7
Employment		
Full time	23	5
Part time/occasional	55	13
Unemployed	196	46
Retired	14	3
Unable to work (disabled)	140	33
Monthly Income		
Less than $1,000	240	56
$1,000—$1,999	97	23
$2,000 +	88	21
Ever incarcerated		
Yes	328	77
No	99	23
Homeless in past 12 months		
Yes	170	40
No	259	60
Currently living with spouse or partner		
Yes	62	15
No	365	85
Currently raising or helping raise child		
Yes	79	18
No	350	82
HIV status		
HIV-positive	200	47
HIV-negative	181	43
Indeterminate/inconclusive	8	2
Never tested	34	8
*Who have you told that you have sex with men?*		
My mother or the woman who raised me	113	35
My father or the man who raised me	50	15
My sibling(s)	100	31
My minister or priest	28	9
Female sex partners	91	28
Heterosexual friends	112	34
Homosexual, bisexual, or transgender friends	203	62
Other family	95	29
My doctor	145	44

Totals may differ due to missing values

The scree plot showed comparable breaks in the scree after 3, 7, and 12 factors. We initially decided to rotate and interpret 7 factors to coincide with the 4 M-GRCS scales, 2 IBHS scales, and our expectation of 1 or 2 IRSS scales. However, only five factors remained when we imposed a minimum factor loading of 0.45 in the rotated solution.

The first and third factors, based on the size of the factor loadings, were defined by subsets of M-GRCS items. The first factor represents both Restricted Emotionality and Discomfort with Affection between Men, and the third factor represents Success/Power/Competition, with one item related to masculine presentation (“One must seem strong to be respected”). Ten of the 13 IBHS items defined the second factor, and IRSS items defined the next two factors, with five and two items, respectively.

Based on the small size of factor loadings, we removed three items from the M-GRCS, three items from the IBHS, and three items from the IRSS from further analyses. Two of the problematic items from the IBHS (“Having sex with other men is not a problem for me” and “I value my sexuality as it is”) and three of the problematic items from the IRSS (“I cannot imagine a loving sexual relationship between two Black men,” “Black men who have sex with men do nothing for Black people,” and “Black homosexual and bisexual men only make it more difficult for black people in general”) were reverse-worded items. Reverse-worded items can be unclear and confusing to respondents (Weijters & Baumgartner, 2012). In addition, we judged the remaining problematic item from the IBHS (“I would not worry about being strictly heterosexual if I could be loved and accepted as a bisexual or homosexual man”) to be poorly constructed. Furthermore, two of the problematic M-GRCS items (“Men who are overly friendly to me make me wonder about their sexual preference” and “Men who touch other men make me uncomfortable”) may have lacked relevance to our sample of behaviorally bisexual men. These items reflect the theme of restricted affection between men. Bisexual men may experience minimal discomfort with expressions of affection to or from other men, especially in discreet settings. Another problematic M-GRCS item (“Moving up the career ladder is important to me”) also lacked relevance to our sample of low-income men because they were not typically in career jobs. In addition, we decided to remove two other problematic M-GRCS items (“I never want to look or seem weak” and “I do not want to seem effeminate, girlish, soft, or womanly”) and one remaining problematic IRSS item (“I wish I could be an open black gay/bisexual/same gender loving man”) from further analyses because they failed to load on any factors in the rotated factor solution. In summary, a total of five M-GRCS items, three IBHS items, and four IRSS items were removed, leaving 30 M-GRCS items, 10 IBHS items, and 7 IRSS items for further analyses.

We repeated the categorical principal axis factoring procedure with ULS extraction and Promax rotation on the final set of 47 items, at which point some of the factor loadings fell between 0.40 and 0.45; however, we retained these items. The scree plot showed a clear break after four factors. The rotated solution with four factors yielded a simple factor structure with nonoverlapping subsets of M-GRCS items defining two factors, the IBHS items defining a single factor, and the IRSS items also defining a single factor (Table 2). The first and third factors in the loading matrix were defined by nonoverlapping subsets of M-GRCS items, reflecting themes of restricted emotionality/discomfort with affection between men and success/power/ competition, respectively. The second factor is an internalized bi/homophobia factor, and the fourth and final factor is the integrated race and sexuality factor.

**Table 2:** Rotated factor loading and Cronbach alpha coefficients in the solution from the categorical principal axis factoring procedure

	Item	Factor1 M-GRCS^a^	Factor2 IBHS^b^	Factor3 M-GRCS^a^	Factor4 IRSS^c^
1	Verbally expressing my love or caring for another man is difficult for me	**.82**	.10	− .23	.02
2	I have difficulty expressing my tender feelings	**.82**	− .03	.00	.01
3	I have difficulty expressing my emotional needs to my partner	**.81**	− .02	.00	.04
4	Talking (about my feelings) during sex is difficult for me	**.74**	− .06	.03	− .05
5	Strong emotions are difficult for me to understand	**.73**	− .07	− .07	.03
6	Affection with other men makes me tense	**.72**	.16	− .16	− .05
7	I have difficulty telling others I care about them	**.69**	− .24	− .04	− .09
8	I am sometimes hesitant to show my affection to men because of how others might perceive me	**.66**	.08	.16	.06
9	I do not like to show my emotions to other people	**.65**	− .04	.19	.03
1	Expressing feelings makes me feel open to attack by other people	**.64**	.09	.04	.18
11	Expressing my emotions to other men is risky	**.64**	.04	.12	.21
12	Hugging other men is difficult for me	**.63**	.06	− .02	− .18
13	Being very personal with other men makes me feel uncomfortable	**.58**	.07	.19	− .18
14	I often have trouble finding words that describe how I am feeling	**.56**	− .01	.25	.00
15	Men should never show their feminine side	**.54**	.00	.23	− .15
16	Telling others about my strong feelings for them is not part of my sexual behavior	**.48**	.10	.07	.02
17	I worry about failing and how it affects my status as a man	**.42**	.05	.32	.23
18	I wish I were not sexually involved with men	− .06	**.85**	.03	.03
19	I don't like being bisexual	.00	**.78**	− .09	− .05
2	I wish that I could develop more sexual desire towards women	.00	**.77**	.10	.06
21	I am uncomfortable being attracted to both sexes	.01	**.72**	− .10	− .01
22	If someone offered me the chance to be completely heterosexual, I would accept it	− .02	**.71**	.01	.07
23	In the past 90 days, I have tried to become more sexually attracted to women	− .01	**.69**	.05	.01
24	I try to avoid personal or social involvement with gay or homosexual men	.09	**.69**	− .03	− .14
25	I would change my sexual preferences if I could	− .07	**.68**	.01	.10
26	I try to avoid personal or social involvement with men who are bisexual or sexually active with both men and women	.00	**.64**	.05	− .27
27	In the past 90 days, I have tried to stop being attracted to men	.14	**.60**	.00	− .08
28	Winning is a measure of my value and personal worth	− .19	.03	**.84**	− .01
29	I strive to be more successful than others	− .10	.00	**.74**	.05
3	Competing with others is the best way to succeed	.03	− .04	**.68**	− .03
31	Being smarter or physically stronger than other men is important to me	.13	.03	**.64**	− .10
32	I like to feel superior to other people	.02	− .05	**.62**	− .19
33	Men must seem strong to be respected	.14	− .02	**.60**	− .09
34	Making money is part of my idea of being a successful man	− .12	.05	**.57**	.30
35	I measure other people's value by their level of achievement and success	.22	− .13	**.52**	− .14
36	Doing well all the time is important to me	.02	.07	**.50**	.29
37	I often feel that I need to be in charge of those around me	.25	− .08	**.50**	− .04
38	I am often concerned about how others judge my performance at work or school	.25	.04	**.47**	.16
39	I sometimes define my personal value by my career success	.14	.13	**.42**	.13
4	It is important for men to look tough	.28	.02	**.41**	− .03
41	A Black man who has sex with men can still be a strong man	− .01	− .08	− .07	**.90**
42	Black homosexual and bisexual men can play an important role in Black families	− .04	− .05	− .08	**.71**
43	Black homosexual and bisexual men contribute to black communities	.04	− .01	− .05	**.61**
44	Both my race and my sexuality are important to who I am as a man	.04	.08	.01	**.61**
45	It is more important that people see me as a man than as a heterosexual man	.10	.03	.01	**.61**
46	It is more important that people see me as a man than as a Black person	− .08	.03	.20	**.52**
47	I know Black men who are in loving committed relationships with each other	− .01	− .17	.02	**.51**
	Cronbach α	*α* = .93	*α* = .89	*α* = .87	*α* = .74

^a^Modified Gender Role Conflict Scale

^b^Internalized Bi/Homophobia Scale

^c^Integrated Race and Sexuality Scale

Table 2 provides internal consistency reliability estimates and inter-factor correlations. Coefficient alphas for the two M-GRCS factors (*α* = 0.93 for Factor 1 and 0.87 for Factor 3) and the IBHS factor (2) (*α* = 0.89) were high and lower but acceptable for the IRSS (*α* = 0.74). As shown in Table 3, both M-GRCS factors were positively correlated with the IBHS factor. In addition, the first M-GRCS factor (Restricted Emotionality/Difficulty with Affection between Men) was negatively correlated with the IRSS factor, *r*(411) = -0.21, while the second M-GRCS factor (Success/Power/Competition) had a weak positive correlation with the IRSS, *r*(412) = 0.09. However, we note that the latter included items related to both general emotionality and to emotion/affection between men. As expected, the IBHS factor was negatively correlated with the IRSS, *r*(414) = -0.22. Most of these correlations are “medium” size based on Cohen’s rules of thumb: 0.10 is small, 0.24 is medium, and 0.37 is large (Cohen, 1988).

**Table 3:** Inter-factor^a^ correlations

	Factor 1 M-GRCS	Factor 2 IBHS		Factor 3 M-GRCS	Factor 4 IRSS
Factor 1	1.0				
Factor 2	.41		1.0		
Factor 3	.50		.31	1.0	
Factor 4	− .21		− .22	.09	1.0

^a^Factor 1 = restricted emotionality/affection. Factor 2 = internalized bi/homophobia. Factor 3 = success/power/competition. Factor 4 = integrated race and sexuality

Our examination of correlations between the new/modified factors and the criterion measures found associations in the expected directions (not shown). The product–moment correlation between the IRSS and the Lukwago Racial Pride Scale was large, *r*(417) = 0.40, *p* < .0001. Among the 323 participants who had told someone that they had sex with men, we found a large negative correlation of the IBHS with the number of friends knowing their sexual orientation, *r* (322) = -0.37, *p* < .0001, and the perceived importance of one’s friends knowing this, *r*(414) = -0.20, *p* < .0001. We also found statistically significant negative, small, point-biserial correlations between IBHS and disclosing to the following types of network members: one’s mother, father, siblings, heterosexual friends, homosexual/bisexual/transgender friends, and doctor (all biserial *r*’s range from -0.13 to -0.21). In addition, we found statistically significant positive correlations between IBHS and the importance of keeping one’s sexual relationships with men secret, *r*(414) = 0.31, *p* < .0001, and between IBHS and having sex with women who did not know one’s sexual orientation, *r*(396) = 0.12, *p* = .02.

Finally, we examined correlations of the scales with well-established measures of self-esteem, psychological distress, and social support (Table 4). In general, we found statistically significant associations in the expected directions. The Rosenberg Self-Esteem Scale was negatively associated with restricted emotionality/affection between men, internalized bi/homophobia, success/power/competition (Factors 1–3) and positively associated with the IRRS (Factor 4). Statistically significant associations were found between all 9 subscales of the Brief Symptom Inventory (BSI) and Factors 1–3. In addition, statistically positive associations were found between the friends and family subscales of the Multidimensional Scale of Perceived Social Support (MSPSS) and the IRSS. However, the MSPSS was largely uncorrelated with the three factors representing the M-GRCS, IBHS, and M-GRCS.

**Table 4:** Correlations of identified factors with self-esteem, psychological distress, and perceived social support

Psychosocial scale	Factor 1 M-GRCS	Factor 2 IBHS	Factor 3 M-GRCS	Factor 4 IRSS
Rosenberg self-esteem	− .34^c,d,*^	− .19^c,d,*^	− .17^c,d,*^	.32^c,*^
BSI^a^ somatization	.16^*^	.12^*^	.13^*^	− .08^c,d^
BSI-obsessive–compulsive	.21^*^	.14^*^	.17^*^	− .05^c,d^
BSI-interpersonal	.18^*^	.10^*^	.16^*^	− .05^c,d^
BSI-depression	.19^*^	.12^*^	.13^*^	− .05^c,d^
BSI-anxiety	.22^*^	.16^*^	.18^*^	− .12^c,d,*^
BSI-hostility	.23^*^	.13^*^	.22^*^	.01^c,d^
BSI-paranoia	.15^*^	.13^*^	.13^*^	− .06^c,d^
BSI-psychoticism	.25^*^	.18^*^	.16^*^	− .05^c,d^
MSPSS^b^-friends	− .2^c,d,*^	− .10^c,d,*^	− .01^c,d^	.23^c,*^
MSPSS-family	− .12^c,d,*^	− .02^c,d^	.02^c,d^	.15^c,*^

Factor 1 = restricted emotionality/affection. Factor 2 = internalized bi/homophobia. Factor 3 = success/power/competition. Factor 4 = integrated race and sexuality

^a^Brief Symptom Inventory

^b^Multidimensional Scale of Perceived Social Support

^c^These are positive outcomes

^d^Items that should be negatively associated with positive outcomes

^*^p < .05

## Discussion

The reliabilities of our modified and new measures were generally high. Furthermore, the correlations with racial pride and disclosure of same-sex sexual support the construct validity of the measures. Additionally, their correlations with self-esteem and psychological distress point to the relevance of the measures to the psychological well-being of Black MSMW. The reliabilities were roughly comparable to prior studies that used the original version of the measures. However, prior research on the original GRCS and IHS from which the IBHS was derived would suggest that we would expect a 6-factor structure from these three scales. In other research on the GRCS over the years, studies with participants from multiple races and specifically with gay men consistently supported a 4-factor, rather than a 2-factor structure (Wester, Vogel, O’Neil, & Danforth, 2012; Zhang et al., 2015).

Our analysis of the reduced and refined set of the Modified Gender Role Conflict Scale and the Internalized Bi/Homophobia Scale and the new Integrated Race and Sexuality Scale items administered to a sample of Black MSMW yielded a four-factor structure consisting of Success/Power/Competition and Restricted Emotionality/Discomfort with Affection between Men factors defined by M-GRCS items, an Internalized Bi/Homophobia factor defined by IBHS items, and an Integrated Race and Sexuality Factor defined by seven of the IRSS items. In addition, the Success/Power/Competition and Restricted Emotionality/Discomfort with Affection between Men factors were positively intercorrelated, but negatively correlated with the IRSS factor.

By using an intersectional framework to modify the measures of gender role conflict and bi/homophobia, we were able to address expectations of masculinity that may place a greater emphasis on sexual prowess, physical dominance, and gamesmanship for Black men and less of an emphasis on education, employment, and socioeconomic status than for White men (Fields et al, 2015; LaPollo et al., 2014). The correlations among our factors indicated that high levels of success/power/competition were associated with high levels of restricted emotionality/affection between men and with high levels of internalized bi/homophobia. We also found that internalized bi/homophobia was associated with a decreased likelihood of disclosure to multiple types of individuals in the men’s social network, to their healthcare providers, and to their female sex partners. These patterns of results support the construct validity of the modified scales.

The M-GRCS appears to behave differently in low-income Black MSMW than it does in other subgroups of men. Although this may be explained by our scale modifications rather than the population focus, it is not surprising that gender-role-related conflicts for economically marginalized Black MSMW differ significantly from those observed in the middle-class White heterosexual and gay-identified men who have been the most studied with the GRCS. For example, Black cultural norms regarding emotional expression and affection between men differ from those of many Whites, with greater acceptance of displays of strong emotion and of public affection between close heterosexual male friends, including treating certain friends as family and sharing the material rewards of success (Oware, 2011). Culturally acceptable displays of gender also differ. An activity such as dancing, for example, was deemed feminine or “gay” by White male high school students, while Black male high school students saw dancing as a way to affirm their masculinity (Miller, 2015). Thus, our intersectional framework and our findings bring into sharp relief how some GRCS items may have less relevance than has been seen with samples from other racial/ethnic groups. Our framework also points to the cultural limitations of recently developed measures of bisexuality for BMSMW (Antebi-Gruszka & Schrimshaw, 2018; Dyar et al., 2019; Paul et al., 2014): they did not include enough BMSMW in their samples, ignored race as a domain, or used terms such as “straight” to refer to people other than the participants—even though some of the BMSMW identified themselves as “straight.”

We were somewhat surprised that the M-GRCS and IBHS were largely uncorrelated with social support despite having negative associations with self-esteem and positive associations with psychological distress. One potential explanation to explore in future research is that adhering to these norms and beliefs reflects alignment with like-minded members of the dominant culture, even though the beliefs have costs at the individual psychological level.

### Limitations

We used the original (unreduced) version of our M-GRCS scale in a publication that examined associations of gender role conflict with psychosocial factors, sexual risk behaviors, and HIV testing (Harawa et al., 2013). For the factors examined in both papers, we found similar associations for the two different versions of the measure. However, the reduced M-GRCS is a more parsimonious and internally consistent measure than the full M-GRCS. Although the wording changes that we made for both the M-GRCS and the IBHS were intended to make the measure more relevant for the target population, we cannot be sure about whether or not they had other unintended effects on the measures’ psychometric properties. Another limitation is the self-selected nature of our sample who self-identified for participation in an HIV prevention intervention and were from a limited geographic area. Finally, in the context of HIV risk behaviors, it is difficult to define and measure gendered psychosocial constructs such as hypermasculinity and sexual orientation without conflating them with labels and behaviors, e.g., MSM. Hence, these findings need to be replicated in other samples.

### Conclusion

The literature does not provide a clear picture of the health impacts of gender role expectations and bi/homophobia on Black men who have sex with men and women. As we have argued, part of the problem is that research has traditionally ignored an intersectional framework for thinking about the identities of Black men in general and Black MSMW in particular. Misunderstandings of the complexity and intersectionality of Black MSMW’s identities may result from differences in the measures used across studies and the use of incommensurate measures for examining gender and masculinity constructs in MSM and MSMW and in Black and White men. From the perspective of HIV prevention, the discrepancies others have observed in the associations of these factors with specific risk and preventive behaviors when studying BMSMW and BMSM or White MSMW corroborate the argument that BMSMW have unique circumstances and racial and cultural contexts that warrant research in their own right (Malebranche, 2008). This study sought to address this by developing an instrument specifically for Black MSMW and examining its psychometric properties in a moderately large sample of men who were defined using a specific but not overly narrow definition of MSMW (any sexual behavior with men within the prior two years). The Integrated Racial and Sexual Identity Scale we developed examines race, gender, and sexuality as joint rather than independent identities. It also takes into account that a narrow focus on gender- and sexual orientation-related attitudes overlooks the ways in which a strong African-American racial identity can contribute to reduced sexual risk behaviors (LaPollo et al., 2014; Li et al., 2018). Although this scale did not perform as well as the modified versions of existing measures, it provides a new tool that can be refined and built upon in future research. This new instrument may contribute to future research in shaping findings on the sexual risk behaviors and the role of masculinity and bi/homophobia in the lives of Black MSM and MSMW.

## References

[CR2] Antebi-Gruszka N, Schrimshaw EW (2018). Negative Attitudes Toward Same-Sex Behavior Inventory: An internalized homonegativity measure for research with bisexual, gay, and other non-gay-identified men who have sex with men. Psychology of Sexual Orientation and Gender Diversity.

[CR3] Bingham TA, Harawa NT, Williams JK (2013). Gender role conflict among African American men who have sex with men and women: Associations with mental health and sexual risk and disclosure behaviors. American Journal of Public Health.

[CR5] Bowleg L, del Río-González AM, Holt SL, Pérez C, Massie JS, Mandell JE, Boone CA (2017). Intersectional epistemologies of ignorance: How behavioral and social science research shapes what we know, think we know, and don’t know about U.S. Black men’s sexualities. Journal of Sex Research.

[CR4] Bowleg L, Teti M, Massie JS, Patel A, Malebranche DJ, Tschann JM (2011). ‘What does it take to be a man? What is a real man?’: Ideologies of masculinity and HIV sexual risk among Black heterosexual men. Culture, Health & Sexuality.

[CR6] Cattell RB (1966). The scree test for the number of factors. Multivariate Behavioral Research.

[CR7] Carey MP, Senn TE, Seward DX, Vanable PA (2010). Urban African-American men speak out on sexual partner concurrency: Findings from a qualitative study. AIDS and Behavior.

[CR8] Cohen J (1988). Statistical power analysis for the behavioral sciences.

[CR9] Cronbach LJ (1951). Coefficient alpha and the internal structure of tests. Psychometrika.

[CR10] Derogatis LR, Melisaratos N (1983). The Brief Symptom Inventory: An introductory report. Psychological Medicine.

[CR11] Dyar C, Feinstein BA, Davila J (2019). Development and validation of a brief version of the Anti-Bisexual Experiences Scale. Archives of Sexual Behavior.

[CR12] Dyer TP, Regan R, Wilton L, Harawa NT, Ou SS, Wang L, Shoptaw S (2013). Differences in substance use, psychosocial characteristics and HIV-related sexual risk behavior between Black men who have sex with men only (BMSMO) and Black men who have sex with men and women (BMSMW) in six US cities. Journal of Urban Health.

[CR13] Estrada F, Rigali-Oiler M, Arciniega GM, Tracey TJ (2011). Machismo and Mexican American men: An empirical understanding using a gay sample. Journal of Counseling Psychology.

[CR14] Feinstein BA, Moran KO, Newcomb ME, Mustanski B (2019). Differences in HIV risk behaviors between self-identified gay and bisexual young men who are HIV-negative. Archives of Sexual Behavior.

[CR15] Fields EL, Bogart LM, Smith KC, Malebranche DJ, Ellen J, Schuster MA (2015). "I always felt I had to prove my manhood": Homosexuality, masculinity, gender role strain, and HIV risk among young Black men who have sex with men. American Journal of Public Health.

[CR16] Ford CL, Whetten KD, Hall SA, Kaufman JS, Thrasher AD (2007). Black sexuality, social construction, and research targeting ‘The Down Low’(‘The DL’). Annals of Epidemiology.

[CR17] Friedman MR, Bukowski L, Eaton LA, Matthews DD, Dyer TV, Siconolfi D, Stall R (2019). Psychosocial health disparities among black bisexual men in the U.S.: Effects of sexuality nondisclosure and gay community support. Archives of Sexual Behavior.

[CR18] Friedman MR, Sang JM, Bukowski LA, Chandler CJ, Egan JE, Eaton LA, Stall R, Stall R (2019). Prevalence and correlates of PrEP awareness and use among Black men who have sex with men and women (MSMW) in the United States. AIDS and Behavior.

[CR19] Guttman L (1940). Multiple rectilinear prediction and the resolution into components. Psychometrika.

[CR20] Hall NM, Applewhite S (2013). Masculine ideology, norms, and HIV prevention among young Black men. Journal of HIV/AIDS & Social Services.

[CR21] Hammond WP, Mattis JS (2005). Being a man about it: Manhood meaning among African American men. Psychology of Men & Masculinity.

[CR24] Harawa NT, Williams JK, McCuller WJ, Ramamurthi HC, Lee M, Shapiro MF, Xx WE, Cunningham WE (2013). Efficacy of a culturally congruent HIV risk-reduction intervention for behaviorally bisexual black men: Results of a randomized trial. AIDS.

[CR22] Harawa NT, Williams JK, Ramamurthi HC, Bingham TA (2006). Perceptions towards condom use, sexual activity, and HIV disclosure among HIV-positive African-American men who have sex with men: Implications for heterosexual transmission. Journal of Urban Health.

[CR23] Harawa NT, Williams JK, Ramamurthi HC, Manago C, Avina S, Jones M (2008). Sexual behavior, sexual identity, and substance abuse among low-income bisexual and non-gay-identifying African-American men who have sex with men. Archives of Sexual Behavior.

[CR25] Hendrickson AE, White PO (1964). Promax: A quick method for rotation to oblique simple structure. British Journal of Statistical Psychology.

[CR26] Herek GM, Gillis JR, Cogan JC, Glunt EK (1997). Hate crime victimization among lesbian, gay, and bisexual adults: Prevalence, psychological correlates, and methodological issues. Journal of Interpersonal Violence.

[CR27] Hunter AG, Davis JE (1992). Constructing gender: An exploration of Afro-American men’s conceptualization of manhood. Gender & Society.

[CR28] Hunter AG, Davis JE (1994). Hidden voices of Black men: The meaning, structure, and complexity of manhood. Journal of Black Studies.

[CR29] LaPollo AB, Bond L, Lauby JL (2014). Hypermasculinity and sexual risk among black and white men who have sex with men and women. American Journal of Men's Health.

[CR30] Li MJ, Frank HG, Harawa NT, Williams JK, Chou CP, Bluthenthal RN (2018). Racial pride and condom use in post-incarcerated African-American men who have sex with men and women: Test of a conceptual model for the men in life environments intervention. Archives of Sexual Behavior.

[CR31] Lukwago SN, Kreuter MW, Bucholtz DC, Holt CL, Clark EM (2001). Development and validation of brief scales to measure collectivism, religiosity, racial pride, and time orientation in urban African American women. Family & Community Health.

[CR32] Malebranche DJ (2008). Bisexually active Black men in the United States and HIV: Acknowledging more than the "Down Low". Archives of Sexual Behavior.

[CR33] Mankowski ES, Maton KI (2010). A community psychology of men and masculinity: Historical and conceptual review. American Journal of Community Psychology.

[CR34] Martin, J. L., & Dean, L. L. (1987). *Ego-Dystonic Homosexuality Scale*. Unpublished manuscript, Columbia University.

[CR35] Mayfield W (2001). The development of an internalized homonegativity inventory for gay men. Journal of Homosexuality.

[CR36] Meyer IH (1995). Minority stress and mental health in gay men. Journal of Health and Social Behavior.

[CR37] Miller B (2015). ‘‘Dude, where’s your face?’’ Self-presentation, self-description, and partner preferences on a social networking application for men who have sex with men: A content analysis. Sexuality & Culture.

[CR38] Nunn A, Dickman S, Cornwall A, Rosengard C, Kwakwa H, Kim D, Xx KH, Mayer KH (2011). Social, structural and behavioral drivers of concurrent partnerships among African American men in Philadelphia. AIDS Care.

[CR39] O’Neil J, Helms B, Gable R, David L, Wrightsman L (1986). Gender Role Conflict Scale: College men’s fear of femininity. Sex Roles.

[CR40] Oware M (2011). Brotherly love: Homosociality and Black masculinity in Gangsta Rap music. Journal of African American Studies.

[CR41] Parks CW (2001). African-American same-gender-loving youths and families in urban schools. Journal of Gay & Lesbian Social Services.

[CR42] Paul R, Smith NG, Mohr JJ, Ross LE (2014). Measuring dimensions of bisexual identity: Initial development of the Bisexual Identity Inventory. Psychology of Sexual Orientation and Gender Diversity.

[CR43] Rhodes SD, Hergenrather KC, Vissman AT, Stowers J, Davis AB, Hannah A, Xx FF, Marsiglia FF (2011). Boys must be men, and men must have sex with women: A qualitative CBPR study to explore sexual risk among African American, Latino, and White gay men and MSM. American Journal of Men's Health.

[CR44] Rosenberg M (1979). Conceiving the self.

[CR45] Shadaker S, Magee M, Paz-Bailey G, Hoots BE, NHBS Study Group (2017). Characteristics and risk behaviors of men who have sex with men and women compared with men who have sex with men-20 US cities, 2011 and 2014. Journal of Acquired Immune Deficiency Syndromes.

[CR46] Szymanski D, Carr E (2008). The roles of gender role conflict and internalized heterosexism in gay and bisexual men’s psychological distress: Testing two mediation models. Psychology of Men & Masculinity.

[CR47] Wade JC, Rochlen AB (2013). Introduction: Masculinity, identity, and the health and well-being of African American men. Psychology of Men & Masculinity.

[CR48] Ward EG (2005). Homophobia, hypermasculinity and the US black church. Culture, Health & Sexuality.

[CR49] Weijters B, Baumgartner H (2012). Misresponse to reversed and negated items in surveys: A review. Journal of Marketing Research.

[CR50] Wester SR, Vogel DL, O’Neil JM, Danforth L (2012). Development and evaluation of the Gender Role Conflict Short Form (GRCS-SF). Psychology of Men & Masculinity.

[CR51] Williams JK, Ramamurthi HC, Manago C, Harawa NT (2009). Learning from successful interventions: A culturally congruent HIV risk-reduction intervention for African American men who have sex with men and women. American Journal of Public Health.

[CR52] Zhang C, Blashill AJ, Wester SR, O’Neil JM, Vogel DL, Wei J, Zhang J (2015). Factor structure of the Gender Role Conflict Scale-Short Form in Chinese heterosexual and gay samples. Psychology of Men & Masculinity.

[CR53] Zimet GD, Dahlem NW, Zimet SG, Farley GK (1988). The Multidimensional Scale of Perceived Social Support. Journal of Personality Assessment.

